# Risk Factors for Clinically Relevant Postoperative Pancreatic Fistula (CR-POPF) after Distal Pancreatectomy: A Single Center Retrospective Study

**DOI:** 10.1155/2021/8874504

**Published:** 2021-01-20

**Authors:** Gao Qing Wang, Dipesh Kumar Yadav, Wei Jiang, Yong Fei Hua, Cai De Lu

**Affiliations:** ^1^Department of Hepatobiliary and Pancreatic Surgery, Ningbo Medical Center LiHuiLi Hospital, Ningbo 315040, Zhejiang, China; ^2^Department of Hepatobiliary Surgery & Liver Transplantation, The First Affiliated Hospital, Zhejiang University, Hangzhou 310003, China

## Abstract

**Objectives:**

Clinically relevant postoperative pancreatic fistula (CR-POPF) is the considerable contributor to major complications after pancreatectomy. The purpose of this study was to evaluate the potential risk factor contributing to CR-POPF following distal pancreatectomy (DP) and discuss the risk factors of pancreatic fistula in order to interpret the clinical importance.

**Methods:**

In this retrospective study, 263 patients who underwent DP at Ningbo Medical Center Li Huili Hospital between January 2011 and January 2020 were reviewed in accordance with relevant guidelines and regulations. Patients' demographics and clinical parameters were evaluated using univariate and multivariate analyses to identify the risk factors contributing to CR-POPF. *P* < 0.05 was considered statistically significant.

**Results:**

In all of the 263 patients with DP, pancreatic fistula was the most common surgical complication (19.0%). The univariate analysis of 18 factors showed that the patients with a malignant tumor, soft pancreas, and patient without ligation of the main pancreatic duct were more likely to develop pancreatic fistula. However, on multivariate analysis, the soft texture of the pancreas (OR = 2.381, 95% CI = 1.271–4.460, *P*=0.001) and the ligation of the main pancreatic duct (OR = 0.388, 95% CI = 0.207–0.726, *P*=0.002) were only an independent influencing factor for CR-POPF.

**Conclusions:**

As a conclusion, pancreatic fistula was the most common surgical complication after DP. The soft texture of the pancreas and the absence of ligation of the main pancreatic duct can increase the risk of CR-POPF.

## 1. Introduction

In recent decades, distal pancreatectomy (DP) has become a common surgical technique for the treatment of benign and malignant pancreatic tumors, chronic pancreatitis, and pancreatic trauma [1]. Technically, DP is a simpler procedure compared to pancreaticoduodenectomy (PD), as a pancreatoenteric anastomosis is seldom required, and prevention of postoperative pancreatic fistula remains a challenge in DP due to an ineffective closure of the pancreatic remnant. The incidence of pancreatic fistula after DP ranges from 5% to 32%, depending upon the definition used and the underlying pancreatic pathology [2–6]. As per updated definition of the International Study Group for Pancreatic Fistula (ISGPF) in 2016, only grade B and grade C postoperative pancreatic fistulas are considered as a clinically relevant postoperative pancreatic fistula (CR-POPF) as it is associated with a clinically relevant development/condition related directly to the postoperative pancreatic fistula, and an earlier grade A postoperative pancreatic fistula is now no longer considered as a true pancreatic fistula because it has no clinical importance, instead it is now reported as a “biochemical leak” [7].

CR-POPF is the considerable contributor to major complications such as bleeding, abdominal abscess, sepsis, and even death following pancreatic resection [8–10]. Nevertheless, various attempts have been made to improve surgical outcomes that include suture closure of the pancreatic stump, staple transection of the pancreas, the use of fibrin glue to cover the pancreatic stump, coverage of the pancreatic stump with autologous tissue, the use of pancreatic stents, and the use of prophylactic octreotide [10]. Woefully, most of these methods have failed to improve fistula rates [10, 11]. Nonetheless, risk identification and risk stratification might benefit in the prevention of POPF. Indeed, the development of the fistula risk score (FRS) for PD and its application has provided a great understanding for the prediction of POPF and has guided the modern accessible mitigation techniques in reduction of morbidity [10, 12]. However, the underlying mechanism of POPF after DP is still poorly understood, and FRS for DP has not been developed yet that can predict the risk of POPF.

The purpose of this study was to evaluate the potential risk factors contributing to CR-POPF following DP and discuss the risk factors of pancreatic fistula to interpret the clinical importance.

## 2. Patients and Methods

### 2.1. Patients

All the patients who underwent DP at the Ningbo Medical Center Li Huili Hospital between January 2011 and January 2020 were reviewed retrospectively from the electronic medical record system. The study was approved by an Institutional Ethical Committee of the Ningbo Medical Center Li Huili Hospital and was consistent with the Declaration of Helsinki [13]. Written informed consent was obtained from all the patients or patients party before the surgery. The present data analysis includes 263 patients (*n* = 213 underwent open distal pancreatectomy and *n* = 50 cases underwent laparoscopic distal pancreatectomy) undergoing DP over a 9-year period. Data were collected from the medical records on the standardized datasheets for all patients, and the variables collected were patients' demographics, surgery indications, preoperative evaluation and risk evaluation, preoperative lab values, perioperative and postoperative course that includes age, body mass index (BMI), smoking, and preoperative American Society of Anesthesiologists (ASA) risk grading [14], indication for surgery, pancreas texture, combined multivisceral resection, splenectomy, ligation of the main pancreatic duct, treatment of pancreatic stump, preoperative diabetes, intraoperative blood loss, use of somatostatin after surgery, preoperative albumin level, postoperative albumin level (3 days after surgery), surgical approach (open vs. laparoscopic), operation time, and pancreatic resection range.

### 2.2. Treatment Protocols

All patients underwent preoperative contrast-enhanced abdominal computed tomography or enhanced magnetic resonance imaging examination with cholangiopancreatography (MRCP) to accurately assess the nature of the lesion, location, size, and the relationship with the splenic vessels and other organs. Additionally, perioperative prophylactic antibiotics and a daily dose of low molecular weight heparin (LMWH) were given to all patients. Moreover, all of the patients also received prophylactic subcutaneous 200 *µ*g of octreotide as an induction dose. Nasogastric (NG) tubes were routinely placed throughout the operation. Furthermore, two tubes were generally placed at the end of an operation for drainage of fluid, i.e., a Jackson-Pratt drain (JP drain) near to the pancreatic stump remnant and another passive drainage tube in the operation field. Simultaneously, postoperative pain was managed by an epidural anesthesia or patient-controlled analgesia (PCA), and all the patients were shifted to the intensive care unit (ICU) for a night. Besides, after surgery, some patients received a continuous intravenous infusion of octreotide at the rate of 0.25 mg/hr for 7 days with the help of a microinfusion pump on the random basis according to the surgeons preference.

Enhanced recovery after surgery (ERAS) protocol was used for postoperative management of all the patients, focusing on early mobilization and early nutrition intake [15]. Additionally, abdominal fluid drainage was monitored, and if the amount of drainage fluid was <10 ml after 24 hrs, the passive-drainage tube was withdrawn after an inspection with ultrasound to exclude any collection of fluid in the abdominal cavity. Moreover, the serum amylase level and drainage fluid amylase level (from JP drain) were examined after 3 days to rule out the presence of pancreatic fistula. In addition to this, at the time of follow-up, Doppler ultrasound was used see the patency of splenic vessels (for the patients with spleen-preserving DP with preservation of splenic vessels) and to rule out any thrombus or stricture in vessels. Furthermore, all the data were documented prospectively in the hospital database.

### 2.3. Operative Techniques

An operation was performed by 3 senior surgeons of our department. Moreover, the choice of surgical technique was decided by consultation between the surgeons and the patient party or according to underlying disease condition on preoperative radiological evaluation.

#### 2.3.1. Surgical Procedure for Laparoscopic DP

Laparoscopic DP was mostly carried out for benign and low-grade malignant tumors in the distal pancreas. The surgical techniques for laparoscopic DP have already been described in detail in our previous studies [16, 17]. After ruling out any other abdominal pathology, metastasis, and any puncture to internal organs, abdominal surface of the pancreas was exposed by dissection of gastrocolic and gastrosplenic ligaments using a laparoscopic harmonic scalpel. Great care was taken to preserve the left gastroepiploic vessels and short gastric vessels. Furthermore, the dissection was performed according to the surgeons preference, and both superior-anterior approach [17] and inferior-posterior approach [16] are being used in our hospital for spleen-preserving DP with preservation of splenic vessels (Kimura technique [18]) by taking advantage of the avascular plain known as “the fusion fascia of Toldt” [16]. Warshaw technique [19] was only performed for low-grade malignant tumor with suspected or known cases of tumor invading the splenic vessels. After obtaining adequate surgical margin and after sufficient mobilization of the pancreas, the pancreas was divided proximally approximately 2 cm far from the tumor with the help of the Covidien Endo GIA Universal Straight 60–3.5 mm stapler. Additionally, in order to free the distal pancreatic stump together with the body and tail from the splenic vessels, it was dissected dorsally with the help of an ultrasonic knife by pulling it to the left lateral side. Furthermore, to minimise the risk of POPF, in the recent years, we routinely suture the pancreatic stump using polypropylene 3-0 intracorporeal interrupted sutures. Nonetheless, splenectomy was performed in case of an inadequate blood supply and outflow obstruction of the spleen. Additionally, taking oncologic principle into consideration, splenectomy was also performed if the tumor lies in close proximity to the splenic hilum [20, 21]. At the end of the operation, the specimen was pulled out using a bag via an enlarged umbilical port-site incision and was sent for histopathology. Besides, the texture of the pancreas was determined by the tactile feedback of the instrument and was reassured after being pulled out from the abdominal cavity. Additionally, the texture of the pancreas was also confirmed from the histopathological report of the specimen after an operation based on the fibrosis grade of the pancreatic tissues. Last, the abdominal cavity was washed with warm water, and a JP drain tube was placed close to the pancreatic stump and a passive-drainage tube in the operation field on the left side through 5 mm port-site incisions.

#### 2.3.2. Surgical Procedure for Open DP

Open DP was carried out both for benign tumor and malignant tumor in the distal pancreas. Open DP was performed with bilateral subcostal or upper midline incision. However, except the incision, other techniques were somewhat similar to laparoscopic DP. Nonetheless, in most of the cases, the transection of the pancreas was not performed with the Endo GIA stapler; the transection of pancreatic parenchyma was performed using the surgical blade, and the main pancreatic duct on the remnant pancreatic stump was ligated using 4-0 or 5-0 polypropylene continuous suture whenever identified. Additionally, the remnant pancreatic stump was also sutured using 4-0 or 5-0 polypropylene continuous suture to avoid any leakage from the branch pancreatic duct. In all the cases of malignant tumor, lymphadenectomy and the excision of the nodal tissues were performed along the common hepatic artery, the left gastric artery, the celiac axis, and along the superior mesenteric vein, including the peripancreatic lymph nodes. Additionally, extended resection along with resection of other visceral organs was performed in any cases of contiguous organ involvement.

### 2.4. Definitions

The severity grading of surgical complications was determined as proposed by Clavien–Dindo classification [22]. Moreover, the postoperative complications such as delayed gastric emptying (DGE), [23] postpancreatectomy hemorrhage (PPH) [24], chyle leak [25], and postoperative pancreatic fistula (POPF) [7] were in accordance with the consensus definition of the ISGPS. Precisely, a CR-POPF was defined as an external fistula with a drain output of any measurable volume of fluid after postoperative day 3 with an amylase level more than 3 times the upper limit, associated with a clinically relevant development/condition related directly to the POPF. Additionally, clinical criteria must be met in order to be considered as true POPF. Since, the earlier grade A POPF is not considered as true pancreatic fistula because it has no clinical importance; therefore, it was reported as a “biochemical leak.” Only POPF grades B and C were placed in the category of CR-POPF. Particularly, grade B was defined as any changes required in the postoperative management; drains either left in place more than 3 weeks or repositioned through endoscopic or percutaneous procedures. Similarly, grade C POPF referred to those POPF that required reoperation or lead to single or multiple organ failure and/or mortality attributable to the pancreatic fistula [7]. Additionally, postoperative mortality was defined as the death within 30 days after surgery or death during the hospital stay [26].

### 2.5. Management of the Pancreatic Fistula

All the cases of POPF were managed by adequate tube drainage of the pancreatic stump, administration of octreotide, antibiotic therapy, irrigation, adjustment drainage of the tube in case of blockage, and gradual withdrawal of the drainage tube whenever the amylase and the lipase levels of the drain fluid were lower than 3 times the serum amylase level, and there was less than 50 ml of fluid per day. Additionally, relaparotomy was performed for patients with grade C POPF. However, there was no standard treatment protocol for the management of POPF.

### 2.6. Statistical Analysis

All the statistical analyses were performed using SPSS 16.0 (IBM Corp., Armonk, NY). Continuous data are reported as a mean ± standard deviation (SD). Categorical data are reported as absolute numbers (*n*). The univariate analysis of risk factors for pancreatic fistula was performed by the *χ*^2^ test, and the multivariate analysis was performed by the multivariate logistic regression model (the backward elimination method) to test the independent risk factors for pancreatic fistula. *P* < 0.05 was considered statistically significant.

## 3. Results

All 263 patients, including 124 males and 139 females, underwent DP at the Li Hui Li Hospital and the Ningbo medical center, Ningbo, between January 2011 and January 2020. The median age of the patients undergoing DP was 58 years (range 17–89 years). Of these 263 patients, 121 (46%) had malignant tumors, and 142 (54%) had benign or low-grade malignant tumors (Table 1). Among total patients, 213 patients underwent open surgery, and 50 patients underwent laparoscopic surgery. The mean operation time was 221 ± 90 minutes (230 ± 95 minutes for laparoscopic surgery and 219 ± 83 minutes for open surgery), and mean blood loss was 375 ± 215 ml. There were 165 cases of combined splenectomy, whereas the spleen was preserved in 98 cases. However, endoscopy was not routinely performed at the time of follow-up; none of the patients suffered from gastric or esophageal variceal bleeding due to spleen-preserving DP. The mean follow-up time was 20.6 ± 2.3 months. Multivisceral resections were carried out in 74 (28.1%) patients (4 patients had more than 2 combined organ resections) that include 32 partial gastrectomy, 8 adrenalectomies, 3 left nephrectomy, 18 partial hepatectomy, and 13 partial small intestine or colon resection (Table 2). Moreover, twenty-one patients had extended pancreatic body and tail resection (i.e., the pancreas was cut to the right side of the portal vein).

### 3.1. Management of Pancreatic Remnant

Mitigation techniques of the pancreatic remnant and resection margin were mainly performed by two techniques in our series, i.e., manual closure using sutures and closure using Endo GIA stapling. Manual closure using sutures was employed in 211 patients (80.2%), whereas Endo GIA stapling was used in 52 patients (19.7%). Of these, ligation of the main pancreatic duct was performed in 174 patients (66.1%) overall. The incidence of CR-POPF was 23.1% in Endo GIA stapling and 18% in manual closure using sutures. However, the result was not statistically significant between the two.

### 3.2. Pancreatic Fistula and Other Complications

The total postoperative complications developed in 38.4% (101/263) patients (i.e., one or more than one complication) that includes 50 cases of pancreatic fistula (19.0%), 10 cases of pulmonary infection (3.8%), 5 cases of abdominal bleeding (1.9%), 4 cases of cardiovascular complications (1.5%), 4 cases of chylous fistula (1.5%), 1 case complicated with biliary fistula, gastric fistula, severe abdominal infection, and renal failure in a trauma patient, which was managed after active treatment (0.4%), and 1 case of bile leakage in a patient with liver resection (0.4%) (Table 3). Among the patients who suffered from POPF, there were 61 cases of the biochemical leak, 48 cases (96.0%) of grade B POPF, and 2 cases (4.0%) of grade C POPF. The average time of hospital stay was 24.6 ± 9.3 days in patients with POPF and 19.8 ± 7.3 days in patients without POPF (*P*=0.025). Similarly, the average time of hospital stay was 20.5 ± 7.5 days in patients with biochemical leak and 31.5 ± 9.2 days in patients with CR-POPF (i.e., grade B and grade C POPF). The result was statistically significant between the two groups (*P*=0.038). Postoperative complications due to CR-POPF occurred in 32 patients (64%) include abdominal infection in 20 cases (40%), delayed PPH in 2 cases (4%), DGE in 5 cases (10%), and surgical site wound infection in 5 cases (10%). Fortunately, no postoperative mortality occurred in our series.

### 3.3. Risk Factors for the Development of CR-POPF

Furthermore, all 263 patients were divided into the CR-POPF group (*n* = 50) and non-CR-POPF group (*n* = 213) based on the occurrence of pancreatic fistula. The factors that might contribute in the development of pancreatic fistula are presented in Table 4. The univariate analysis of 18 factors showed that the patients with a malignant tumor, soft pancreas, and patients without ligation of the main pancreatic duct were more likely to develop pancreatic fistula. The incidence of CR-POPF in patients with malignant tumor was 30/121 (24.8%) and 20/142 (14%) in patients with benign disease or low-grade malignant tumors, *P*=0.027. Similarly, the incidence of CR-POPF in patients with the soft pancreas was 25/88 (28.4%) and that of the firm pancreas was 25/175 (14.2%), *P*=0.006. Likewise, the incidence of CR-POPF in patients without ligation of the main pancreatic duct was 26/89 (29.2%) and in patients with ligation of the main pancreatic duct was 24/174 (13.8%). *P*=0.003. However, univariate analysis demonstrated no significant relationship between CF-POPF and the following factors: age, BMI, smoking, ASA, combined multivisceral resection, splenectomy, pancreatic stump treatment, preoperative diabetes, intraoperative blood loss, use of somatostatin after surgery, preoperative albumin level, postoperative albumin level (3 days after surgery), surgical approach (open vs. laparoscopic), operation time, and pancreatic resection range. Only a significantly important association was demonstrated between CF-POPF and the following factors: pancreatic pathology (malignant tumor vs. benign disease or low-grade malignant tumor: 24.8% vs. 14%, *P*=0.027), pancreas texture (soft vs. firm: 28.4% vs. 14.2%, *P*=0.006), and ligation of the main pancreatic duct (no vs. yes: 29.2% vs. 13.8%, *P*=0.003).

Multivariate analysis was performed by the multivariate logistic regression model (the backward elimination method) for all 18 factors used in the univariate analysis. The results showed that the soft texture of the pancreas (OR = 2.381, 95% CI = 1.271–4.460, *P*=0.001) and the ligation of the main pancreatic duct (OR = 0.388, 95% CI = 0.207–0.726, *P*=0.002) were independent influencing factors for CR-POPF (Table 5). The ligation of the main pancreatic duct was associated with lesser number of CR-POPF in the univariate analysis.

## 4. Discussion

In this study, we have examined both mortality and morbidity related to DP, with a particular aimed to POPF. Data from our study showed that DP can now be performed very safely without mortality. However, the higher rate of the morbidity still remains the concern, which was close to 38.4% in our series. Particularly, CR-POPF was the most frequent complication that occurred in 19% of our patients, and the rate of CR-POPF in our series is similar to that reported in the literature [27–29]. Nonetheless, CR-POPF is the considerable contributor to major complications such as peripancreatic effusion, peripancreatic abscess, pseudocyst formation, or erosion and digestion of surrounding tissues, resulting in intraabdominal hemorrhage and gastric emptying disorders, resulting in prolonged hospitalization time and increased hospitalization costs, affecting subsequent treatment following pancreatic resection [7–10]. Additionally, some patients may be readmitted after discharge due to the above complications. In our study, the length of hospital stay for the CR-POPF group was significantly longer than that of the non-CR-POPF group, with postoperative complications due to CR-POPF occurred in 32 patients (64%) including bleeding.

On univariate analysis, CR-POPF occurred significantly at a higher rate in the soft pancreas (vs. the hard pancreas), and on the other hand, CR-POPF occurred significantly at a lower rate in the patients with benign disease or low-grade malignant tumor and when intraoperative ligation of the main pancreatic duct was not performed. No other factors were found to be related to an increased risk of CR-POPF. However, on multivariate analysis, only the texture of the pancreas and the ligation of the main pancreatic duct were independent influencing factors for CR-POPF.

Many studies have revealed various preoperative, intraoperative, and postoperative variables as the risk factors for the development of CR-POPF, i.e., age, intraoperative blood loss, soft texture of the pancreas, BMI, multivisceral resections, splenectomy, operation time, gland thickness, and the fasting blood glucose level [27, 30–32]. However, most of these studies have been inconsistent with their findings with each other. The reasons for the inconsistent findings might be retrospective nature of the studies, heterogeneous practices among the surgeons, and the consequences of a learning curve for CR-POPF occurrence and management in different centers. Thus, the relationship between different risk factors for the development of CR-POPF should be interpreted cautiously. Nonetheless, in most of the studies, soft texture of the pancreas has widely been recognized as the most significant risk factor for the development of CR-POPF [12, 27, 32]. In our series, 88 patients had soft pancreatic texture (CR-POPF 28.4%), and 175 patients had a hard pancreatic texture (CR-POPF 14.3%). Indeed, univariate analysis revealed there were significant statistical differences for the development of CR-POPF between the two groups (soft pancreatic texture vs. hard pancreatic texture), *P*=0.001, attributing that the patients with soft pancreatic texture were more prone to develop a CR-POPF after DP than patients with a hard pancreatic texture. Additionally, multivariate analysis implied that a soft pancreatic texture was an independent risk factor associated with CR-POPF (OR = 2.381, 95% CI = 1.271–4.460, *P*=0.001). The lower rate of CR-POPF in patients with hard pancreatic texture may be explained by pancreatic fibrosis resulting into the exocrinal dysfunction of the pancreas. However, there are yet no standardized criteria to define the texture of the remnant pancreas as “soft” or “hard.” In most of the cases, the texture of the remnant pancreas “soft” or “hard” is determined according to surgeon's own experience during the operation and confirmed by the histopathological report of the specimen after an operation based on the fibrosis grade of the pancreatic tissues. In recent years, some studies have proposed pancreatic elastography as an effective tool for the assessment of the pancreatic remnant texture and maybe used to predict POPF [33, 34]. Moreover, a recent meta-analysis found that a lower strain value on ultrasound shear wave elastography was significantly associated with CR-POPF. A lower strain value on ultrasound shear wave elastography implies to a softer pancreatic tissue. Thus, suggesting that the strain value on ultrasound elastography can be useful as an objective and quantifiable method to assess the pancreatic texture [35]. However, a recent prospective study by Marasco G. et al. concluded that pancreatic ultrasound elastography is not useful in predicting pancreatic fistula after pancreatic resection [36]. Nevertheless, the result of ultrasound elastography of the pancreas and the strain value could be influenced by various factors, such as the difference between ultrasound elastography equipment, the difference in ultrasound elastography techniques, steatosis of the pancreas, pulsations in the artery, obese patients, and the presence of gas in the bowel gas just in front of the pancreas [35]. Consequently, to address this controversy, well-designed prospective studies with a larger sample size are required for evaluating the accuracy of ultrasound elastography in the prediction of CR-POPF.

At present, the main mitigation strategies for the pancreatic remnant to reduce the risk of POPF include manual closure using sutures, closure using ENDO-GIA stapling, the use of fibrin glue to cover the pancreatic stump, coverage of the pancreatic stump with autologous tissue, the use of pancreatic stents, and the use of ultrasonic dissector [10, 27]. Nonetheless, whether these mitigation strategies can reduce or prevent the occurrence of POPF is still debatable. There are several retrospective studies [27, 37], randomized controlled trials (RCTs) [4, 11, 38], and meta-analysis [39, 40] evaluating these mitigation strategies and found no evidence that these techniques are able to prevent or reduce risks of developing CR-POPF. Results from our study suggest that the intraoperative ligation of the main pancreatic duct can reduce the incidence of POPF; this observation was consistent with previous studies [41–43]. In our study, the incidence of CR-POPF was 29.2% when there was no intraoperative ligation of the main pancreatic duct and 13.8% when there was intraoperative ligation of the main pancreatic duct. Nevertheless, elective ligation of the main pancreatic duct might be difficult sometimes, especially when the main pancreatic duct is too thin, and it is difficult to be identified. To overcome such difficulties, we suggest sharp and careful transection of the pancreatic body or tail, where the main pancreatic duct can easily be identified in most of the cases. However, we should also acknowledge that only ligation of the main pancreatic duct is not an ultimate solution for POPF; the opening of the small branch ducts on the margin of the pancreatic remnant may also cause POPF. Because of the contractile resistance of the sphincter of Oddi, the pressure of the main pancreatic duct increases, which results in the formation of POPF due to the opening of the accessory branched pancreatic ducts on the pancreatic remnant. Thus, manual closure using sutures on the margin of the remnant pancreatic stump may be necessary. However, POPF can easily occur in the soft pancreas due to cutting and tearing of pancreatic tissue by sutures. Furthermore, if the suture is densely placed on the pancreatic remnant, it may cause ischemic necrosis of the tissue in the remnant pancreatic stump. Similarly, if the suture is placed too loose, it will cause POPF due to the incomplete suturing of the pancreatic stump. Thus, surgeons must take these factors into consideration while suturing the main pancreatic duct and the remnant pancreatic stump. However, some authors believe that the ligation of the main pancreatic duct does not affect the occurrence of pancreatic fistula [27, 29]. It has been reported that preoperative endoscopic pancreatic stent implantation can effectively reduce the pressure of pancreatic exocrine ducts, thereby reducing the occurrence of pancreatic fistula [44]. Additionally, more recently, a study by Ecker et al. reported that the use of epidural analgesia was associated with significantly fewer incidences of POPF, probably because it is able to reduce the sphincter of Oddi pressure [27]. On the other hand, some authors believe that POPF can effectively be reduced by anastomosis of the pancreatic stump to the stomach (pancreaticogastrostomy) [45] or to the jejunum (pancreaticojejunostomy) [46] after DP [47]. However, the accuracy of these additional operations to prevent POPF remains to be further confirmed, but these additional surgical procedures undoubtedly will increase the complexity of the operation and prolong the time of the operation. In other words, this may increase the possibility of other postoperative complications. For the internal drainage of the pancreatic stump, the authors believe that, if preoperative imaging or intraoperative exploration reveals obstruction of the proximal pancreatic duct, the pancreatic stump should be anastomosed with the jejunum or the posterior gastric wall to drain the pancreatic juice, which may prevent POPF caused by the proximal pancreatic duct pressure.

Endo GIA stapling is a common method in DP for closure of the pancreatic stump, especially for laparoscopic surgery. It has advantages that it can save operation time and can be performed easily compared to the transection of pancreatic parenchyma using the surgical blade. However, there are some unfavourable factors, such as an inadequate ligation of the main pancreatic duct and tension at the edge of the pancreatic stump, which aggravate local ischemia and necrosis of the pancreatic stump. In our series, the incidence of CR-POPF in the Endo GIA stapling group was 23.1% and 18% in the suture group. However, there was no significant difference between both the groups. The reason we speculate for this is that, in the recent years, we routinely suture the pancreatic stump using polypropylene 3-0 intracorporeal interrupted sutures after Endo GIA stapling. Thus, this might have influenced the incidence of CR-POPF in the Endo GIA stapling group. Therefore, we believe that manual suture still remains the mainstream method for the treatment of the pancreatic stump after DP.

Our study has several limitations that need to be emphasized. First, this study is a retrospective nature and thus, subject to biases. Likewise, the data included in this study are over a long period of time (2011–2020) and may have different surgical techniques and POPF mitigation strategies depending upon individual surgeon preference. Similarly, there might be a potential misgrading of patients with biochemical leakage before the updated definition of ISGPS 2016. Second, some clinical data are not sufficient like we could not collect proper data for pancreatic thickness, where different studies have outlined it as an independent risk factor for CR-POPF [48, 49]. Third, the effects of a learning curve on POPF occurrence and management of POPF cannot be excluded. Finally, our electronic medical record system might not have the record of complications that were managed in local hospitals. However, on the other hand, our study is still of great importance, as it includes large size of the cases from a single center. Moreover, we have analyzed most of the clinically relevant variables that might have an effect on the occurrence of POPF in both open and laparoscopic DP.

## 5. Conclusion

Pancreatic fistula was the most common surgical complication after DP. The soft texture of the pancreas and the absence of ligation of the main pancreatic duct can increase the risk of CR-POPF. No other factors such as age, BMI, smoking, ASA, combined multivisceral resection, splenectomy, pancreatic stump treatment, preoperative diabetes, intraoperative blood loss, use of somatostatin after surgery, preoperative albumin level, postoperative albumin level (3 days after surgery), surgical approach (open vs. laparoscopic), operation time, and pancreatic resection range had an influence on development of CR-POPF after DP.

## Figures and Tables

**Table 1:** Primary lesions in 263 patients undergoing pancreatectomy.

Primary lesion	Number of cases	Percentage (%)
Pancreatic cancer	85	32.3
Pancreatic solid-pseudopapillary tumor	9	3.4
Other organ malignancy (gastric cancer and lymphomas invading the pancreas)	25	9.5
Pancreatic trauma	12	4.6
Intraductal papillary mucinous neoplasm (IPMN)	35	13.3
Mucinous cystic neoplasm (MCN)	23	8.7
Pancreatic serous cystadenoma	22	8.4
Pancreatic cystadenocarcinoma	5	1.9
Primary pancreatic non-Hodgkin's lymphoma	4	1.5
Pancreatic pseudocyst	15	5.7
Pancreatic abscess	2	0.8
Pancreatic neuroendocrine tumors	12	4.6
Pancreatic neuroendocrine carcinoma	2	0.8
Intrapancreatic accessory spleen	1	0.4
Chronic pancreatitis	11	4.2

**Table 2:** Multivisceral distal pancreatectomy without the spleen (*n* = 74).

Organ (procedure)	Number
Partial gastrectomy	32
Adrenalectomy	8
Left nephrectomy	3
Partial hepatectomy	18
Small intestine or colon resection	13

Note: 4 patients had more than 2 combined organ resections.

**Table 3:** Postoperative complications.

Complication	*N* (%)
Pancreatic fistula	50 (19.0%)
Pulmonary infection	10 (3.8%)
Abdominal bleeding	5 (1.9%)
Cardiovascular complications	4 (1.5%)
Chylous fistula	4 (1.5%)
Biliary fistula, gastric fistula, severe abdominal infection, and renal failure in a trauma patient	1 (0.4%)
Bile leakage in a patient with liver resection	1 (0.4%)

**Table 4:** Univariate analysis of risk factors for postoperative pancreatic fistula after distal pancreatectomy (DP).

Variables	Number of cases, *n* = 263	Pancreatic fistula, *n* = 50	Nonpancreatic fistula, *n* = 213	*χ* ^2^	*P* value
Age					
≥70	45	9	36	0.034	0.853
＜70	218	41	177		

BMI (kg/m^2^)					
>25	84	11	73	2.806	0.094
≤25	179	39	140		

Smoking					
Yes	67	13	54	0.009	0.925
No	196	37	159		

ASA					
I	114	21	93	0.046	0.831
II-III	149	29	120		

Indication for surgery					
Benign disease or low-grade malignant tumors	142	20	122	4.866	0.027
Malignant tumors	121	30	91		

Preoperative diabetes					
Yes	42	7	35	0.178	0.673
No	221	43	178		

Preoperative albumin level					
≥35 g/L	227	45	182	0.711	0.399
＜35 g/L	36	5	31		

Surgical approach					
Laparoscopic	50	10	40	0.014	0.907
Open	213	40	173		

Operation time					
≥300 min	54	10	44	0.011	0.918
＜300 min	209	40	169		

Pancreas texture					
Soft	88	25	63	7.586	0.006
Hard	175	25	150		

Pancreatic resection range					
Left side of portal vein	242	45	197	0.341	0.559
Right side of portal vein	21	5	16		

Splenectomy					
Yes	165	29	136	0.593	0.441
No	98	21	77		

Combined multivisceral resection					
Yes	70	13	57	0.012	0.913
No	193	37	156		

Ligation of main pancreatic duct					
Yes	174	24	150	9.094	0.003
No	89	26	63		

Pancreatic stump treatment					
Suture	211	38	173	0.696	0.404
Endo GIA stapler	52	12	40		

Intraoperative blood loss					
≥600 ml	46	9	37	0.269	0.604
＜600 ml	217	41	176		

Use of somatostatin after surgery					
Yes	75	14	61	0.067	0.796
No	188	36	152		

Postoperative albumin level (3 days after surgery)					
≥35 g/L	177	34	143	0.001	0.973
＜35 g/L	86	16	70		

BMI, body mass index.

**Table 5:** Multivariate logistic regression analysis for postoperative pancreatic fistula after distal pancreatectomy (DP).

Variables	*β*	S.E.	Wald	*P* value	OR	95% CI
Pancreas texture (soft vs. hard)	−1.116	0.351	10.108	0.001	2.381	1.271–4.460
Ligation of the main pancreatic duct (yes vs. no)	−1.021	0.333	9.407	0.002	0.388	0.207–0.726

*β,* regression coefficient; S.E., standard error of regression coefficient; Wald, Wald chi-square value; CI, confidence interval; OR, odds ratio.

## Data Availability

The data used to support the findings of this study are included within the article and are available from the corresponding author upon request.
